# Multigene phylogenetic analysis redefines dung beetles relationships and classification (Coleoptera: Scarabaeidae: Scarabaeinae)

**DOI:** 10.1186/s12862-016-0822-x

**Published:** 2016-11-29

**Authors:** Sergei Tarasov, Dimitar Dimitrov

**Affiliations:** Department of Research and Collections, Natural History Museum, University of Oslo, P.O. Box 1172, Blindern, NO-0318 Oslo Norway

**Keywords:** Dung beetles, Scarabaeinae, Scarabaeidae, Model adequacy, Classification, Molecular phylogeny

## Abstract

**Background:**

Dung beetles (subfamily Scarabaeinae) are popular model organisms in ecology and developmental biology, and for the last two decades they have experienced a systematics renaissance with the adoption of modern phylogenetic approaches. Within this period 16 key phylogenies and numerous additional studies with limited scope have been published, but higher-level relationships of this pivotal group of beetles remain contentious and current classifications contain many unnatural groupings. The present study provides a robust phylogenetic framework and a revised classification of dung beetles.

**Results:**

We assembled the so far largest molecular dataset for dung beetles using sequences of 8 gene regions and 547 terminals including the outgroup taxa. This dataset was analyzed using Bayesian, maximum likelihood and parsimony approaches. In order to test the sensitivity of results to different analytical treatments, we evaluated alternative partitioning schemes based on secondary structure, domains and codon position. We assessed substitution models adequacy using Bayesian framework and used these results to exclude partitions where substitution models did not adequately depict the processes that generated the data. We show that exclusion of partitions that failed the model adequacy evaluation has a potential to improve phylogenetic inference, but efficient implementation of this approach on large datasets is problematic and awaits development of new computationally advanced software. In the class Insecta it is uncommon for the results of molecular phylogenetic analysis to lead to substantial changes in classification. However, the results presented here are congruent with recent morphological studies and support the largest change in dung beetle systematics for the last 50 years. Here we propose the revision of the concepts for the tribes Deltochilini (Canthonini), Dichotomiini and Coprini; additionally, we redefine the tribe Sisyphini. We provide and illustrate synapomorphies and diagnostic characters supporting the new concepts to facilitate diagnosability of the redefined tribes. As a result of the proposed changes a large number of genera previously assigned to these tribes are now left outside the redefined tribes and are treated as *incertae sedis*.

**Conclusions:**

The present study redefines dung beetles classification and gives new insight into their phylogeny. It has broad implications for the systematics as well as for various ecological and evolutionary analyses in dung beetles.

**Electronic supplementary material:**

The online version of this article (doi:10.1186/s12862-016-0822-x) contains supplementary material, which is available to authorized users.

## Background

With over 6200 described species [1] and a global distribution, dung beetles of the subfamily Scarabaeinae (Coleoptera: Scarabaeidae) provide important ecosystem services [2]. They are one of the primary utilizers of mammalian dung on Earth and are historically one of the most recognized and best researched groups of beetles [1, 3–5]. Recently, dung beetles have also become an established model group in ecology and developmental biology (e.g. [6, 7]). However, a robust classification and phylogenetic hypothesis for dung beetles is not available despite many recent phylogenetic efforts [1, 8–13]. As a result interpretation of their evolutionary, ecological and developmental features is often limited to select species and large scale comparative analyses are practically impossible.

The currently accepted classification splits the Scarabaeinae dung beetles into 12 tribes which, over the last two decades, have been the subject of 16 molecular- and morphology-based phylogenetic studies [1, 8–22]. These studies were reviewed in detail by [1, 7].

The results of most of these studies can be characterized by three common trends. 1) They resolve early branching clades or shallow nodes well, but intermediate nodes remain poorly resolved and/or weakly supported. 2) Seven tribes are always recovered as monophyletic or nearly monophyletic (e.g. Onthophagini, Oniticellini), while three tribes (Deltochilini, Ateuchini, and Coprini) are always polyphyletic. The polyphyletic tribes Deltochilini (=Canthonini) and Ateuchini (=Dichotomiini) together comprise ca. 55% of the total generic diversity in this group. Their highly polyphyletic concepts render the tribal classification in the entire subfamily extremely artificial. 3) The results of these key studies often propose conflicting hypotheses [1] leading to a lack of consensus on dung beetle evolutionary history.

One morphological [1] and two molecular phylogenies [11, 22] can be singled out due to their large taxon sample size and global biogeographic coverage; the rest of the studies are usually limited in these respects. The global morphological phylogeny of [1] comprises all main biogeographic and taxonomic lineages and provides an integrative pattern of phylogenetic relationships in dung beetles largely supported by previous publications. However, that study also stresses the need for more data, primarily molecular, to corroborate its findings.

The two available global molecular phylogenies [11] and [22] are similar in composition of genetic markers (*COI, 16S, 28S* and *COI, 16S, 28S, 12S* respectively) as well as species used. mtDNA markers are known to be saturated by fast evolution and not very informative about relationships above the species level, while the *16S*, *28S* and *12S* rDNA markers are challenging to align and analyze with traditional substitution models. These mitochondrial and rDNA genes are good candidates for resolving shallow divergences but they are less informative for recovering higher-level relationships [23] which calls for assembling larger datasets to improve the robustness of phylogenetic inference.

In this paper, we reconstruct the phylogeny of dung beetles using a molecular dataset that comprises 547 terminal taxa and 8 gene regions. This is the largest dung beetle molecular dataset assembled to date, and includes a large quantity of newly sequenced data. In addition, the present dataset has a global biogeographic coverage and incorporates major phylogenetic lineages and enigmatic taxa. To infer the phylogeny we employed a wide range of analytical approaches including direct optimization (POY), maximum likelihood (ML) and Bayesian inference (BI). The traditional substitution models used in model-based methods (ML and BI) have been frequently shown to poorly reflect the reality of the evolutionary process [24, 25]; thus, their application can be inadequate for some molecular datasets. In this study, we explicitly test for model adequacy using Bayesian posterior assessment [25, 26] and perform partition selection based on the adequacy of the selected models. Although data selection guided by Bayesian posterior assessment allows inferring some meaningful relationships absent in datasets where it was not used, the results of both were generally similar. The efficient application of data selection using model adequacy assessment to large datasets, as the one used herein, is presently difficult due to the lack of computationally advanced software. We conclude that the development of such software can, in future, boost progress of Bayesian posterior assessment methods in phylogenetics.

Our results identify new lineages and corroborate some relationships inferred by earlier studies [1, 10–13, 19, 21]. The consistency of clades between the molecular phylogeny presented here and the most recent morphology based analyses [1] enables us to define new systematic concepts for the highly polyphyletic tribes Dichotomiini, Deltochilini and Coprini. Over the last half-century the concepts of these tribes have been constantly changing because clear synapomorphies which could ensure their unequivocal identification have always been missing. Given the principle of monophyly, we limit these tribes substantially to accommodate only those genera which are closely related to their respective type genera. We use the synapomorphies identified by the global morphological phylogeny of [1] to provide an effective identification of these tribes within their new definitions. Many genera hitherto considered members of these tribes are now excluded from them. We treat those genera as *incertae sedis* and discuss the necessary steps towards their phylogeny-based classification. We also expand the concept of the tribe Sisyphini by adding the genus *Epirinus* that was previously placed in Deltochilini.

## Methods

### Taxon sample and vouchers

A total of 530 specimens of dung beetles (Scarabaeinae) belonging to 137 genera from all 12 tribes and biogeographic regions were sampled. 95 specimens from 72 species were sequenced specifically for this study. Representatives of the following dung beetle genera are sequenced for the first time: *Haroldius, Canthonella, Cryptocanthon, Homocopris, Leotrichillum, Paracanthon, Paraphytus, Scatimus, Tesserodoniella,* and *Trichillidum*. The outgroup comprised 17 terminals belonging to 10 genera from the Scarabaeidae subfamilies Chironinae, Aegialiinae and Aphodiinae which are closest relatives of Scarabaeinae based on previous studies [27–31]. Accession numbers and other relevant vouchers information is summarized in Additional file 1: Table S1. In all figures, tables, and Additional files, specimens sequenced for this study are marked with * next to their species names. List of genera with author citations is given in Additional file 2: Table S2.

In this study the tribal classification for genera follows [7]; nomenclature for family-group names follows [32] and [33]. Along with traditional concepts for some tribes in the discussion we also propose newly circumscribed concepts, which are marked as *sensu novo*. The name Ateuchini is used according to [32] to address genera conventionally treated as Dichotomiini (see also "Changes in classification" section) and the name Deltochilini is used as a senior synonym for Canthonini [32].

The voucher specimens used in this study are deposited as indicated in Additional file 1: Table S1. Abbreviations used in the tables are as follows:

- *CEMT:* Seção de Entomologia da Coleção Zoológica, Departamento de Biologia e Zoologia, Universidade Federal de Mato Grosso, Cuiabá, Brasil (F. Vaz-de-Mello).
- *UPSA* University of Pretoria, Insect collection (C. Deschodt and C. Scholtz).
- *ZMUC* Natural History Museum of Denmark (A. Solodovnikov and S. Selvantharan).
- *CNCI* Canadian National Collection of Insects, Arachnids and Nematodes, Ottawa (V. Grebennikov and B. Gill).
- *ABTS* Andrew Smith private collection, Canada, Ottawa.
- *NZAC* New Zealand Arthropod Collection, Auckland (R. Leschen and S. Forgie)
- *ZMUN* Natural History Museum, Oslo, Norway (V. Gusarov).
- *ANIC* Australian National Insect Collection, Australian Capital Territory, Canberra City, CSIRO, (C. Lemann and T. Weir)

### Molecular markers

We used 8 phylogenetically informative markers: 16S ribosomal RNA (*16S*), 18 s ribosomal RNA (*18S*), 28S ribosomal RNA domain 2 (*28SD2*), 28 s ribosomal RNA domain 3 (*28SD3*), cytochrome c oxidase I (*COI*), carbamoylphosphate synthethase (*CAD*), topoisomerase I (*TP1*) and wingless (*Wg*). Mitochondrial (both rDNA and protein encoding) and the nuclear rDNA genes have been widely used in previous studies of dung beetles [11–13, 18–21] and represent the bulk of data for this group in GenBank. Only three phylogenetic studies focused on Africa and Madagascar have used nuclear protein-coding genes *CAD* and/or *TP1* [12, 21, 34]. In this study, we use the nuclear gene *Wg* for the first time in a dung beetle study along with the rDNA regions (*18S, 28SD2, 28SD3*) and *CAD, TP1*. We combine our new sequence data with the data from the same markers available in GenBank (total: 547 terminals, alignment length 5837 bp) to address higher-level relationships of dung beetles (Additional file 3: Matrix S1) .

### DNA extraction, PCR amplification, and sequencing

Genomic DNA was extracted from the head and/or prothorax or legs, following the Qiagen DNeasy Blood & Tissue Kit (QIAGEN) tissue protocol. PCR follows [35] with the following modifications: the reaction was performed in a 20 μL reaction volume using, 0.5 μM of each primer, 10 μL AmpliTaq Gold, Master Mix (Applied Biosystems), and 3 μL of the respective genomic DNA extract. If target genes were difficult to amplify 0.4 μg Bovine Serum Albumin (BSA) were added. The general PCR profile consisted of an initial denaturation step at 94 °C for 2 min, followed by 30 cycles at 94 °C for 1 min, 52–68 °C for 30 s, and 72 °C for 1-2 min, and a final extension step of 10 min at 72 °C. The annealing temperature was optimized separately for each pair of primers. *TP1, CAD, Wg* were amplified using the nested PCR approach described by [36]. All primers used for amplification and amplification strategies are listed in Additional file 4: Table S3. The PCR products were purified with ExoSAP-IT (Stratagene), and then sequenced. All fragments were sequenced in both directions. The GenBank accession numbers of the sequences are given in Additional file 1: Table S1.

### Sequence alignment and secondary structure prediction

The sequences were managed, edited and assembled into contigs, and the contigs arranged into the final datasets in Geneious version R6 [37].

For the phylogenetic analyses, alignments were performed with the web-based version of MAFFT [38] (http://mafft.cbrc.jp/alignment/software/) using Q-INS-i option, that takes into account secondary structure, for rDNA genes with less than 300 sequences (*18S, 28SD2*), and L-INS-i for the rest. The secondary structure for rDNA genes was reconstructed in RNAalifold [39] based on the alignments obtained from MAFFT.

Simultaneous alignment and structure prediction for Bayesian model adequacy assessment was performed using LocArna [40]. The size of datasets that can be operated by LocArna is limited to 30 sequences which make this method inapplicable for large phylogenetic analyses. In order to make computations feasible we reduced the dataset by randomly selecting a set of 30 sequences of each gene for model adequacy analyses to fit LocArna requirements. Simultaneous reconstruction and alignment in LocArna better fits our purpose for the detailed exploration of partitions despite the necessary dataset reduction.

### Selection of sites, sequences and partitioning

#### Site and sequence selection

The 3^rd^ codon positions of *COI* were excluded from all analyses (hereafter addressed as the dataset “ALL”) as they have been suggested to suffer saturation for deep divergences which can potentially bias phylogenetic analyses (e.g., [41, 42]). For some analyses, sites containing gaps in more than 20% of the sequences were also removed (dataset “G20”). The value of 20% was found empirically as an optimal trade-off between removing gap-rich sites capable of potentially introducing noise and, at the same time, keeping a sufficient amount of the original sites for the phylogenetic inference. Finally, for the last set of analyses, in addition to the previously removed sites, we also removed the six partitions which yielded low p-values in Bayesian posterior prediction (dataset “DT3”, see Results: Model adequacy section). In total all datasets comprised ~40% of missing data due to incomplete sequencing, their alignment lengths were 5838 bp, 4775 bp and 4016 bp for ALL, G20 and DT3 datasets respectively.

In order to test sensitivity of the incomplete sequencing, we also composed two reduced datasets consisting of species for which at least 4 and 5 genes were assembled (244 and 77 species respectively). Each reduced dataset was also analyzed using maximum likelihood method with different portions of sites excluded (i.e. ALL, G20 and DT3).

#### Partitioning

Initially, the entire dataset was split into 28 *a priori* data blocks. This was done based on the secondary structure (loops and stems regions) for each rDNA gene and based on domain structure and codon position for each protein-coding gene. The domain structure was obtained from InterPro database [43, 44] using Geneious InterProScan plugin v. 1.0.6.

We used Partition Finder [45] under Bayesian Information Criterion (BIC) and the greedy algorithm option in order to find the best partitioning scheme and models. To partition the data for the phylogenetic analyses, we ran Partition Finder on the dataset from the MAFFT analysis using the 28 *a priori* data blocks and 200 randomly selected sequences to reduce computational time. The searches were performed on the set of models implemented in MrBayes excluding a subset of invariant site models, as using the invariant site and the gamma parameter at the same time is not advisable ([46], the RAxML v8.1.X Manual).

Partition Finder analyses of the 28 *a priori* data blocks (run #1) found best partitioning scheme comprising 19 partitions (536 parameters, BIC = 192851.786103). In this scheme, loop and stem region of rDNA genes were placed in a separate partition whereas protein-coding genes were partitioned by codon position and gene. Since this partitions number was still high and could result in computational issues, we manually partitioned the rDNA genes in only two partitions (stem and loop regions) and concatenated some partitions of the protein coding genes mainly based on codon positions. This reduced the number of partitions from the 19 inferred partitions to 10. Partition Finder was run again (run #2) on the data set with 10 partitions resulting in a better BIC score (487 parameters, BIC = 163834.428304) and a scheme retaining the 10 partitions as initially set (Additional file 5: Table S4). The failure of Partition Finder to find the 10-partition scheme from the beginning (or any better partitioning than the proposed 19 partitions) is likely a shortcoming of the greedy algorithm. The scheme from run#2 and the one with the best BIC score were used in the ML analyses.

In the tests of model adequacy, the Partition Finder was run separately for each gene on its respective *a priori* data blocks from the LocArna alignment results.

### Model adequacy assessment

The model adequacy assessment on big datasets, as the one used in the present study, is limited by the software capacity designed for such analyses and the lack of necessary computational pipelines. Thus, as a proxy to model adequacy, we randomly selected a set of 30 sequences for each gene (see Sequence alignment section). Each gene aligned in LocArna was then split into its *a priori* data blocks and run separately in Partition Finder to test for the best partitioning scheme and models (Additional file 6: Table S5). To test models adequacy we used Bayesian posterior assessment (BPA) as implemented in PuMA [47]. Each inferred partition, after excluding sites containing gaps (since PuMA cannot handle gaps) was separately analyzed in MrBayes (see Maximum likelihood and Bayesian inference section) to sample parameters from the posterior distribution. The sampled parameters were used to perform BPA in order to test whether the selected model can adequately capture the process which generated the analyzed sequences.

### Maximum Likelihood (ML) and Bayesian Inference (BI)

Both BI and ML analyses were run on the High Performance Computing cluster Abel at USIT, the University of Oslo.

#### ML

The ML analyses were run in RAxML version 8.0.26 [46] using the three different datasets ALL, G20 and DT3 and the partitioning scheme from Partition Finder run#2 (Additional file 5: Table S4, and Site selection and partitioning section). We used *–f a* option to perform rapid Bootstrap analysis (1000 replicates) and search for best scoring ML tree in one program run the GTRCATX model *(-m GTRCATX)* applied to each partition; the final tree was evaluated under GTRGAMMA model.

#### BI

For the purpose of testing model adequacy, we ran MrBayes using the default priors and the following options: *ngen = 5 M, samplefreq = 5 K, nchains = 4*, and *temp = 0.2*.

Bayesian phylogenetic inference was performed in MrBayes version 3.2.2. [48] and ExaBayes version 1.4.1 [49] using ALL, G20 and DT3 datasets. Both programmes MrBayes and ExaBayes use similar analytical procedure. ExaBayes in contrast to MrBayes implements only GTR models and exponential prior for branch length (unlike compound Dirichlet priors in MrBayes). At the same time, ExaBayes provides advanced parallelization and computational techniques that significantly speed up computations in comparison to MrBayes.

For the actual phylogenetic analyses, we ran MrBayes with default priors except for the branch length. The default exponential branch length prior is known to cause bias in the branch length estimates in partitioned datasets [50, 51]. We used the compound Dirichlet prior instead as suggested by [52] and [51]. The full description of the analysis set-up is provided in the Additional file 7.

In ExaBayes we ran only unpartitioned analyses under the GTR model to avoid biased estimation of branch length due to the use of exponential branch length prior in partitioned data [50, 51]. For each dataset (G20 and DT3) the two runs in ExaBayes were ran with default priors and one heated chain (heatFactor 0.3) for 100 M generations, sampling parameters every 1000^th^ generation. The two runs converged after 50 M which were discarded as burn in. Sdsf between the runs dropped below the acceptable value of 5% being 0.022 and 0.018 for G20 and DT3 dataset respectively.

### Direct optimization (POY)

For the direct optimization analyses protein coding genes were treated as preealigned while ribosomal genes were split into homologous regions based on amplicon limits and preliminary MAFFT alignments. This procedure was necessary because many sequences were missing some of the amplicons or had areas with poor quality that were excluded in the process of sequence editing, resulting in length variation that is not due to insertions/deletions. Limits of different regions were marked with # and matrices were analyzed under maximum parsimony direct optimization. Direct optimization analyses were carried out in the computer program POY v 5.1.1b [53]. We used a search strategy based on iterated timed searches (multiple Wagner trees followed by SPR + TBR + ratchet and tree fusing) for 4–6 h as described in [54]. The strategy uses a series of timed searches that take, as an input, the best tree from the previous round until results stabilize and further iterations consistently find the same trees. There are large numbers of potential combinations of insertion/deletion, gap extension and substitution costs that can be explored in POY. Here we selected a limited number of parameter schemes that have been shown to perform optimally in other studies or have been suggested as best on philosophical grounds. For example the parameter set 3221 (indel opening cost = 3; indel extension cost = 1; transversions = transitions = 2), was suggested as best using philosophical reasoning by J De Laet [55]. The parameter sets investigated were: 111, 121, 211, 221, 3221 and 3211.

## Results

### Model adequacy

**Fig. 1:**
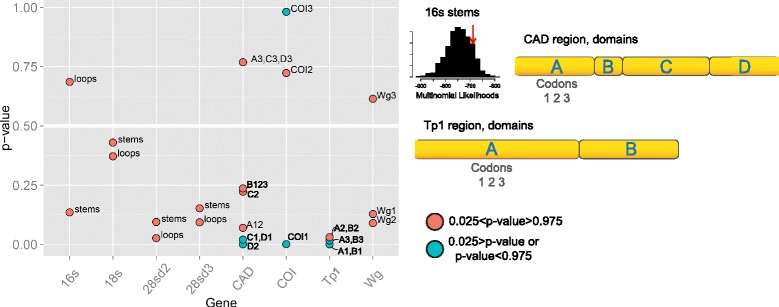
Partitions and model adequacy assessment. Left graph shows per partition *p*-values for every gene. The p-values test a null hypothesis that model applied to partition is adequate based on multinomial test statistics in PuMA (histogram for the *16* *S* gene on the top exemplifies multinomial test statistics). Partitions with values within the 95% two-tailed confidence interval are shown with red circles (null hypotheses is supported), while those with values outside the tails of the distribution are blue circles (they are excluded from dataset DT3). *P*-value approaching 0.5 correspond to highest model adequacy. Partitions consist of a priori data blocks based on secondary structure (rDNA genes), codon position (COI and Wg) or codon position and domain structure (CAD, Tp1). In data blocks names the capital letter corresponds to domain (shown on the right) while number indicate codon position. Additional information and domain names are given in Additional file 6: Table S5

Results from the assessment of model adequacy are summarized in Fig. 1. The posterior predictive *p*-values for the majority of the partitions fall within the 95% confidence interval (Fig. 1, red circles) indicating that models used to analyze these data adequately capture (to a certain extent) the process of their evolution. For this analysis the highest model adequacy corresponds to partitions with p-value approaching 0.5 whereas the models with extremely high or low values in this two-tailed test should be rejected. Interestingly, all rDNA genes demonstrate *p*-values that were not significantly different from our null-model and *18S* shows the best performance amongst all markers used in the present study. Unreasonable model specifications were found only in some protein-coding genes partitions, with *TP1* generally showing the worst scores (*p*-values < 0.05, Fig. 1, blue circles).

### Phylogenetic analyses

The full and two reduced datasets of at least 4 and 5 genes (e.g. Additional files 8 and 9: Tree S12-13) yielded similar topologies and support values but the reduced datasets did not recover some well-corroborated groups found here and in previous studies as they were lacking more than 50% of terminals present in full dataset. Given the significance of taxon sample size in assessing global phylogeny, we limit our discussion below only to the datasets based on the full taxon sample.

**Fig. 2:**
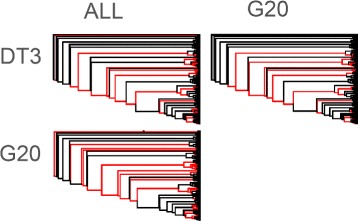
Pairwise comparison of ML trees between analyses with ALL, G20 and DT3 dataset. Branches that differ between analyses are colored in red

**Fig. 3:**
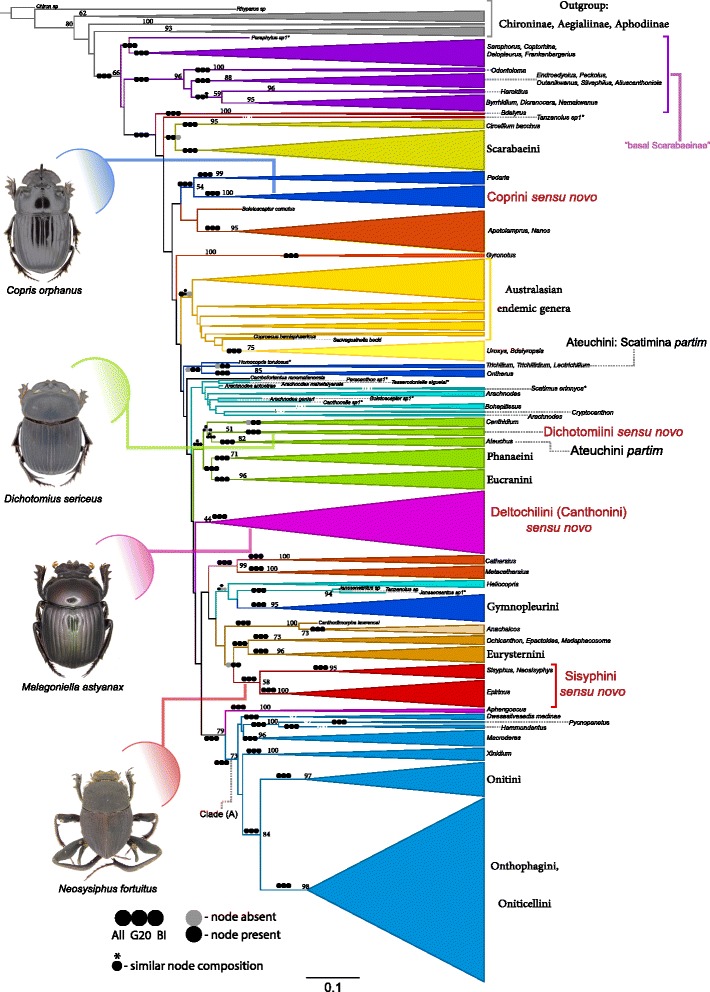
Maximum likelihood tree of Scarabaeinae. ML tree of 547 Scarabaeinae terminals and outgroup. The tree shown here is from the analyses of the DT3 dataset. *Black* and *grey circles* mapped onto branches of this tree indicate presence/absence of node (clade) in ML analyses with ALL and G20 datasets as well as Bayesian analysis (BI). Similar but not identical node (clade) composition is marked with * above *black circle*. The majority of terminals are cartooned based on taxonomy, with the size of the cone corresponding to the number of analyzed terminals. The color of braches is used for readability purpose. Values above branches indicate bootstrap support that is shown only if value > 50%. Representative taxa are shown for the revised tribes discussed in this study

All datasets (ALL, G20, DT3) analyzed using ML produced congruent topologies that differed mainly in linkage of intermediate branches (Figs. 2, 3, Additional files 10, 11 and 12: Tree S1-3). The percentage of shared clades between any two of three datasets was high (ALL & G20 = 74.9%, ALL & DT3 = 72.5%, G20 & DT3 = 75.8%, Additional file 13: Table S7).

The results from the MrBayes analyses were not satisfactory – standard deviation of split frequencies (0.077) was higher the acceptable value of 0.05. Nevertheless, the inferred consensus tree can be, to certain extent, considered stable (see Additional file 7 for more details). Poor convergence in MrBayes is known to occur when analyzing big datasets [56, 57] due to ineffective MCMC sampling from the posterior distribution of topologies [58].

Despite the BI convergence issues, results from ML and BI analyses were generally also congruent (percentage of shared clades with any of three ML analyses ranges from 71.1% up to 75.5%, Additional file 13: Table S7). However, the partitioned Bayesian analysis in MrBayes (Additional files 14 and 15: Tree S4, S5) was more similar to the ML topologies when compared with the unpartitioned analysis from ExaBayes. Because of this higher incongruence and less reasonable partitioning scheme (single partition) we do not overview the ExaBayes results in detail. The partitioned Bayesian analysis is also congruent to ML results in terms of support for intermediate branches, many of which are unresolved in the Bayesian consensus tree from the partitioned analysis and vary among ML analyses depending on the dataset. At the same time, both ML and BI trees were drastically different from the POY trees (Additional files 16, 17, 18, 19, 20 and 21: Tree S6-S11). POY yielded trees with many genera and well-supported monophyletic groups appearing as polyphyletic. Because results from POY were highly divergent from any other published phylogeny and from ML and BI analyses performed here (see Results) we did not continue with further exploration of the results under direct optimization.

Since all ML and BI (in MrBayes) analyses are similar, for illustration purposes we select the ML analysis of the DT3 dataset as a base topology. This is also the dataset with the highest number of inadequate partitions excluded. Then, in order to summarize the results from the other two datasets and the congruence among analyses, we mapped the same clades onto the DT3 tree (Fig. 3, Additional file 12: Tree S3). The differences and similarities in major lineages among the analyses are further summarized in a greater detail in Additional file 22: Table S8.

Monophyly of Scarabaeinae was supported by all ML and BI analyses. Also, all ML analyses recover “basal Scarabaeinae” as paraphyletic lineage whose side branch leads to all the remaining Scarabaeinae. Almost all genera were recovered monophyletic with just a few of them (i.e. *Heliocopris*, *Tanzanolus, Janssensantus, Boletoscapter, Arachnodes, Canthidium,* and *Frankebergerius*) poly- or para-phyletic depending on the dataset analyzed.

The majority of tribes in their conventional definitions emerge monophyletic except for Oniticellini that is nested within Onthophagini, and the highly dispersed Deltochilini, Ateuchini (Dichotomiini) and Coprini. For the latter three tribes new concepts are established (see Systematic entomology section).

In our phylogeny, *Paraphytus* and *Haroldius* are placed within the basal Scarabaeinae: the most basal taxon *Paraphytus* is sister to *Sarrophorus*-like genera, while *Haroldius* is a sister to *Byrrhidium + Dicranocara + Namakwanus* clade. African *Pedaria* comes up as sister to *Copris + Litocopris* (Fig. 3).

The endemic Madagascan genera formerly treated as Deltochilini split into four lineages that are spread across the tree: (i) genera *Apotolamprus + Nanos* form a separate clade (ii) genus *Epactoides* emerges in the same clade with Oriental *Ochicanthon* and Afro-Madagascan *Madaphacosoma* (ii) *Arachnodes* comes up monophyletic in results of the G20 dataset while it is polyphyletic in results based on the DT3 dataset and (iv) genus *Cambefortantus* forms a separate lineage; in results from the G20 dataset it is sister to the Australian *Boletoscapter*.

In ML analyses, all Australasian endemic genera except *Boletoscapter* tended to form a paraphyletic lineage with the Neotropical genera *Uroxys + Bdelyropsis* nested within it.

The neotropical tribe Eurysternini is sister to the Afro-Madagascan-Oriental clade formed by the genera *Madaphacosoma*, *Ochicanthon* and *Epactoides*. A large monophyletic group (clade A) is composed of taxa with primarily Old World origin (e.g. [59, 60]). It includes the tribes Onitini, Onthophagini, Oniticellini along with the genera *Xinidium*, *Macroderes*, *Hammondantus* and *Pycnopanelus*.

South African deltochiline-like *Epirinus* is recovered as sister to the primarily Old World genera *Neosisyphus* and *Sisiphus* traditionally assigned in the tribe Sisyphini.

The American tribes Phanaeini and Eucranini are recovered as monophyletic and sister to the other American genera from the tribe Ateuchini/Dichotomiini (namely, *Canthidium, Dichotomius* and *Ateuchus*).

The neotropical Ateuchini subtribe Scatimina [61] splits into two lineages, one includes the genera *Trichillum*, *Trichillidium* and *Leotrichillum* and the other is comprised by *Scatimus*. However, the ML analysis of the ALL dataset and both Bayesian analyses supports sister relationship between *Scatimus* and *Ateuchus*.

Only DT3 dataset recovered monophyly of genera *Canthidium, Heliocopris* and *Frankebergerius* and close relationships between *Homocopris* and *Ontherus*.

Some noteworthy groups were not recovered in the DT3 and ALL datasets; however, they were recovered by the ML analyses of the less data restrictive G20 dataset. A clade including the Neotropical genera *Tesserodoniella*, *Homocopris,* and *Paracanthon* was resolved. The African genus *Gyronotus* appears as a close relative of the African clade *Anachalcos + Canthodimorpha*. Finally the Neotropical *Canthonella* was nested within Australasian endemics clade.

## Discussion

### Data, model adequacy and partitions

#### Bayesian Posterior Assessment (BPA)

The traditional model selection procedure in phylogenetics focuses on selecting the best model from a set of substitution models using statistical criteria such as AIC, BIC, Bayes factor, etc. However, this procedure does not guarantee that the selected model can be reasonably applied to the data due to factors such as heterogeneous evolutionary rates or selection, which can violate assumptions of the available substitution models. Use of substitution models that do not adequately capture the evolutionary processes in the data may in turn result in biased phylogenies. It has been suggested that testing for model adequacy should be an important step in phylogenetic analysis, although it remains poorly explored and rarely implemented [25, 26].

One of the ways to test the model adequacy is to use posterior predictive assessment (BPA) in a Bayesian framework [25, 26, 62, 63]. The BPA applied, in this study [47], uses a sample of parameters from the posterior distribution of the Bayesian analyses to simulate molecular datasets and then, assesses the probability of seeing the original dataset in the array of the simulated ones based on the multinomial likelihood test statistics. Our results show that not all molecular partitions that we initially designed were adequate in BPA framework (e.g., *TP1*). Use of such datasets or data partitions with available substitution models (even with the models found to fit the data best under AIC or BIC for example) do not adequately capture the process that generated the data and may lead to biased results.

We also show that BPA performance substantially differs between codon and domain position in protein-coding genes. While some parts of these genes can be adequately analyzed with the traditional phylogenetics models, others may have to be excluded from phylogenetic analyses. This finding further stresses the need for choosing an appropriate partitioning scheme and assessment of model adequacy prior to the phylogeny estimation.

All ML and BI analyses produced similar topologies sharing 71–75% of identical clades (Additional file 13: Table S7). The analyses including partitions that performed poorly under the BPA test (ALL and G20) did not differ significantly from the results of the DT3 dataset that excludes all partitions that did not pass the BPA test. This points to a strong phylogenetic signal in the part of the data where substitution models did perform plausibly.

Selecting and testing data using BPA has a statistically solid basis [25, 26, 62, 63] and brings a great potential to improve phylogenetic inference. However the current implementation of this approach to big datasets, as the one used here, is problematic due to the lack of software capable to perform efficient computations on big datasets. The large size of our dataset did not allow implementing BPA analysis in a Bayesian framework under partitioned scheme (in MrBayes for example). The alternative program ExaBayes, that provides high level of parallelization and computational speed, is currently lacking a proper conjugate prior (e.g. compound Dirichlet prior) for tree branch length, which may bias the analyses when using data partitioning. Thus, at present, large datasets can be efficiently analyzed only in ML framework using RaxML program that uses exclusively GTR model for phylogenetic inference, thereby providing a limited model choice for the inference and BPA procedure.

#### Partition scheme search

In addition to use of BPA as a tool to evaluate data and model performance we also used the program Partition Finder in order to select optimal partition scheme for the analyses. Here we used the program following the manual recommendations, i.e. providing an initial set of partitions and letting the algorithm find the best partitioning scheme. However, we found that this procedure may not necessarily find the best solution (as measured by BIC score). We show that, at least in the present case, it is possible to further improve partition schemes by manually altering the results from Partition Finder. Identification of the reason for this behavior was beyond the scope of this study, although it is presumably due to the use of the greedy algorithm option. Therefore, we strongly encourage researchers relying on this algorithm to follow a procedure as the one outlined in the Methods section.

### Dung beetles higher level relationships

#### Trees and analyses

Many of the clades supported by the present phylogeny are consistent with previous phylogenetic treatments of dung beetles [10–13, 19, 21]; and, the present results are also highly congruent with the global morphological phylogeny [1]. This similarity between studies shows that results from different sources tend to converge on an underlying pattern in enlarged datasets. The differences across datasets and ML and BI analyses were insignificant in the context of higher-level relationships. Exclusion and inclusion of different partitions had its advantages and disadvantages; some meaningful relationships inferred in the first case were absent in the second and vice versa. This is likely a result of the heterogeneous nature of the evolutionary process that influences the performance of a marker across a given tree. Although the excluded partitions are found by the BPA as inflicting potential bias on phylogenetic inference, they can be locally informative, especially in resolving recent divergences. The exclusion of these partitions may result in data deficiency at that level and decreased resolution for shallow nodes.

POY trees show significant differences from ML and BI trees and all other published phylogenies. We hypothesize, that this odd behavior of POY in the present study is probably result of the large portion of missing data (~40%), which negatively affects the direct optimization method.

#### Molecules vs. Morphology

The position of “basal Scarabeinae” with *Paraphytus*, as was initially predicted by morphology [1, 10], is largely congruent with present molecular results. The position of *Haroldius* as sister to *Byrrhidium + Dicranocara + Namakwanus* within “basal Scarabaeinae” is surprising but strongly supported (see bootstrap values for the preceding ancestor nodes). The phylogenetic affiliations of *Haroldius* have long remained enigmatic: preliminary morphological analysis placed it in Onthophagini [64], while other authors placed it within Deltochilini [65].

The Australasian clade found here is similar to that supported by morphological analysis (clade Aus1, Fig. 6 in [1]), although in both cases endemic Australasian genera do not form a strictly monophyletic group. Interestingly, morphology, unlike molecules, strongly supports Australian *Boletoscapter* within Australasian clade (Aus1 in [1]); whereas this study recovers neotropical *Uroxys + Bdelyropsys* nested within Australasian clade. Alternatively, another molecular [22] phylogeny suggests sister relationships between *Uroxys + Bdelyropsys* and *Boletoscapter* but does not support such Australasian clade. African *Pedaria*, having significant morphological similarities was recovered within Aus1 by morphology [1] and a previous molecular study [11]; however, in the present study it is placed as a sister of Coprini *sensu novo*.

In this study, clade A, comprising some taxa of Old World origin, is moderately supported and biogeographically well defined. In morphology, this clade is split into three remotely related lineages (arrowed clade, part of L2 and K1, Fig. 6 in [1]). It is noteworthy that morphological analyses [1, 10] did not support a sister or close relationship between Onitini and Onthophagini + Oniticellini, which is recovered by molecular phylogenies (e.g. [8, 11]) including the present study. A lack of synapomorphies that would support this grouping in the morphological dataset is likely the cause for this incongruence.

Madagascan *Apotolamprus* and *Arachnodes* form a clade in the morphological phylogeny (clade G1, Fig. 6 in [1]) due to their significant similarities; however, in the present study they appear to not be closely related, which confirms the results of other molecular study [13].

The relationship between the Ateuchini type genus *Ateuchus* and the Ateuchini subtribe Scatimina varies depending on the dataset. The present molecular data suggests that the subtribe Scatimina may be polyphyletic as it is split into two groups *Trichillum* + allied genera and *Scatimus. Scatimus* shows close relationship to *Ateuchus* but that is not the case for the clade including *Trichillum* + allied genera. This contradicts two morphological phylogenies [1, 10], which recover the monophyly of *Ateuchus* + Scatimina, although it is supported only by one homoplastic synapomorphy – presence of trochantofemoral pit [1].

The present results along with previous morphological [1] and molecular [66] studies support the position of deltochiline-like *Epirinus* within the tribe Sisyphini. Based on these results, here we place *Epirinus* in the tribe Sisyphini *sensu novo*. Further arguments for that decision are provided in the "Changes in classification" section below.

The congruence between results from previous molecular analyses [11–13, 19–22], recent morphological analysis [1, 10, 15] and the molecular analysis presented here for the tribes Deltochilini, Ateuchini and Coprini as well as the high support for the sister relationships between *Epirinus* and Sisyphini motivated us to reevaluate the limits of these tribes.

### New tribal concepts and perspectives for new classification

Natural tribal classification for dung beetles is essential to study their diversity, ecology and evolution. Strong polyphyly of some historic tribes found in the present analyses and in previous phylogenies [1, 11–13, 19–22] indicates that the tribal classification as currently defined does not reflect natural units and has to be revised.

Systematic classification must fulfill two main purposes (i) classify the diversity under study into monophyletic units reflecting their evolutionary history and (ii) provide characters that allow unambiguous diagnosability of all included taxa. Given that requirements for monophyly and diagnosability must be fulfilled, splitting a phylogenetic tree into groups (e.g. tribes) is a somewhat subjective procedure – groups can be defined at shallower nodes producing many monophyletic lineages with few terminal taxa or at deeper nodes resulting in fewer groups that include more terminal taxa. In order to comply with the aforementioned classification purposes, the scarabaeine tribes seem to be better defined at more terminal nodes resulting in a somewhat larger number of tribes. At that level molecular and morphological phylogenies are largely congruent and clades are defined by large numbers of synapomorphies. These two properties guarantee well-supported monophyly and efficient identification for the resulting groups. Contrary to that, defining groups at deeper nodes would yield fewer poorly corroborated tribes that are hard to diagnose, because at this level nodes are often supported by single homoplastic synapomorphy. Thus, splitting and not lumping seems to be an efficient way for the development of a new higher level dung beetle classification due to the lack of diagnosability at deeper nodes. Although, in our results some intermediate nodes are still poorly supported, they are irrelevant for the development of new classification as they lack diagnosability in the context of morphology.

Traditional concepts [7] for the tribes Deltochilini, Ateuchini (Dichotomiini) and Coprini render them largely polyphyletic. The results presented here and in the recent morphological phylogeny [1] are consistent in supporting the monophyly of the clades that contain the type genera of those tribes (or tribes considered their synonyms) and their close relatives providing a solid basis for the revision of these tribal concepts (a special case of Coprini is discussed below). Moreover, global morphological analysis [1] identified synapomorphies that allow easy identification and diagnosis of these revised tribes. Until now identification of many dung beetle tribes has been practically impossible because traditional concepts were not based on synapomorphies or diagnostic characters but rather used authors’ intuition and overall habitus similarity. Explicit concepts and clear characters defining the revised tribal definitions presented here contribute to the stability of the dung beetle classification. However, at the same time they leave 101 genera, previously placed in Ateuchini (Dichotomiini), Deltochilini and Coprini, without tribal affiliation (*incertae sedis,* Additional file 23: Table S9). Many of these *incertae sedis* genera cannot be placed easily in other existing tribes (Fig. 3) and it is possible that new taxa will have to be defined in order to accommodate them.

We also propose to expand the concept for the tribe Sisyphini. Sisyphini traditionally comprised only three genera. Both molecular and morphological analyses support sister relationship between the traditional Sisyphini genera and the South African genus *Epirinus* that was formerly placed within the tribe Deltochilini. Monophyly of this new group is supported by three synapomorphies and by high bootstrap and posterior probability in the corresponding analyses. Based on this evidence we propose to transfer *Epirinus* in Sisyphini.

### Changes in classification

#### Tribal concepts

The new limits (*sensu novo*) for tribes proposed here are based on the present results and are also supported by the findings from several recent molecular phylogenetic analyses [11–13, 19–22] and on the global morphological phylogeny [1]. The traditional concepts for the tribe Dichotomiini, Deltochilini (Canthonini), Coprini and Sisyphini follow [7]. List of genera included in the new concept of each tribe (*sensu novo*) is given in Table 2. The family-group names follow [32, 33]. The concepts *sensu novo* for the tribes Deltochilini and Dichotomiini correspond to their concepts *sensu stricto* in [1]. The redifined tribes emerged monophyletic in all the analyses presented here and are also suppotreted by previous phylgoentic work [1, 11–13, 19–22]; their support values are provided in Table 3.

In this study, unlike [32, 33], we consider Dichotomiini and Ateuchini to be different tribes (see “Tribe Dichotomiini *sensu novo* and the case of Ateuchini” section). Our new concept for Dichotomiini introduces changes in the composition of genera in Ateuchini. The list of putative Ateuchini genera is given in Additional file 23: Table S9. The genera, which are, excluded form the revised tribes and treated as *incertae sedis* are also listed in the Additional file 23: Table S9.

#### Tribal diagnoses

**Fig. 4:**
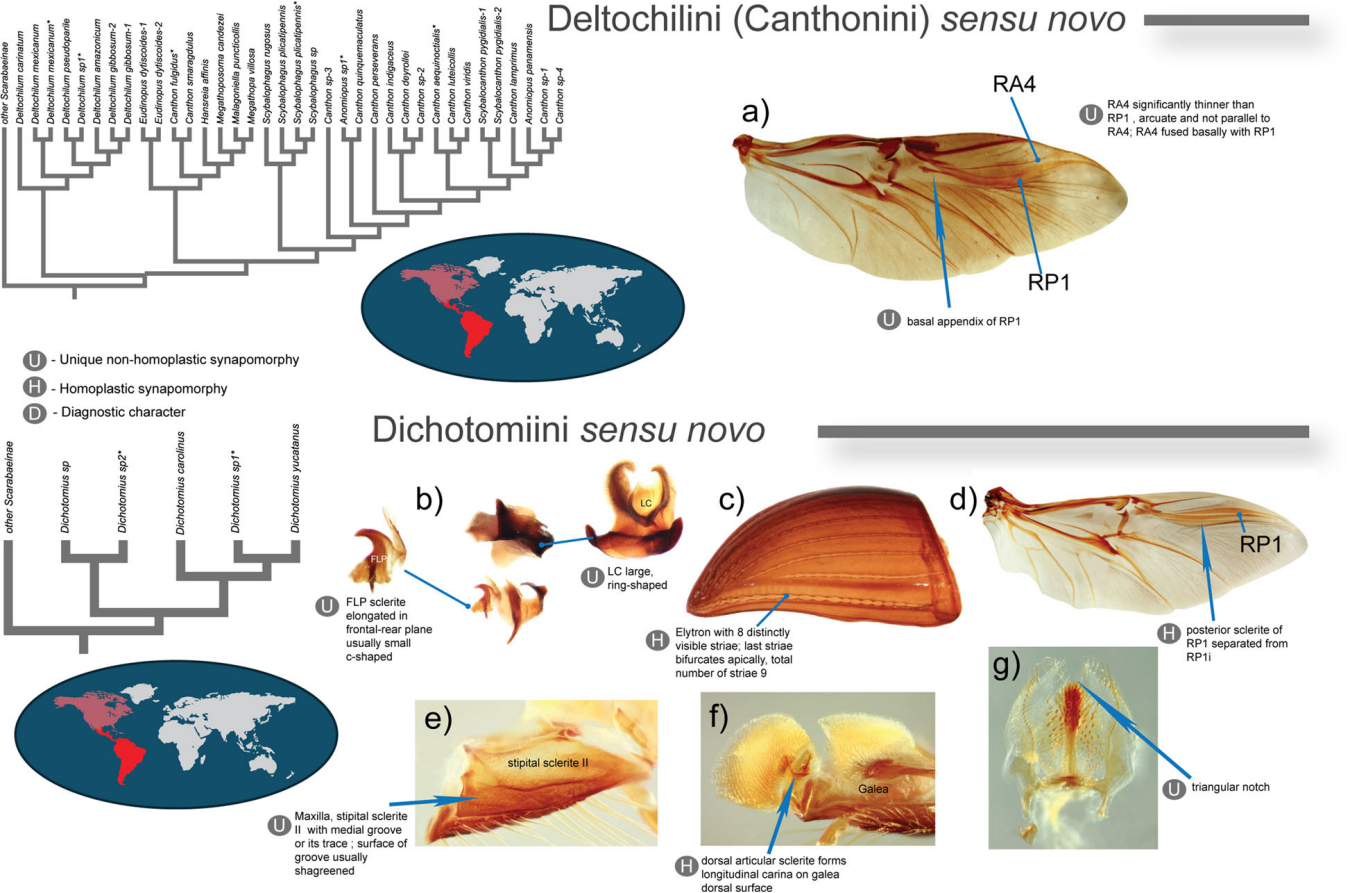
Illustrated synapomorphies and diagnostic characters defining Deltochilini *sensu novo* and Dichotomiini *sensu novo*. Every synapomorphy or diagnostic statement is preceded by a grey circle indicating whether the synapomorphy is unique (U), homoplastic (H), or the statement is diagnostic (D). Explanatory text for character statements is shown next to the images in the figure; additional information is available in Table 1. Vein names are shown for some wing veins for annotation purposes. In some cases morphological parts of species from other tribes are used for illustration purposes. Phylogenetic trees refer to the representatives of the respective tribes from Fig. 3. Maps show the distribution of the tribes per biogeographic region; red color saturation corresponds to approximate species number. **a**). *Canthon virens*; **b**, **e**, **f**, **g**). *Chalcocopris hesperus*; **c**). *Uroxys epipleuralis*; **d**). *Dichotomius sericeus*; **a**, **d**). wing; **b**). aedeagal sclerites; **c**). elytron; **e**, **f**). maxilla; **g**). epipharynx; j, n). prothorax

**Fig. 5:**
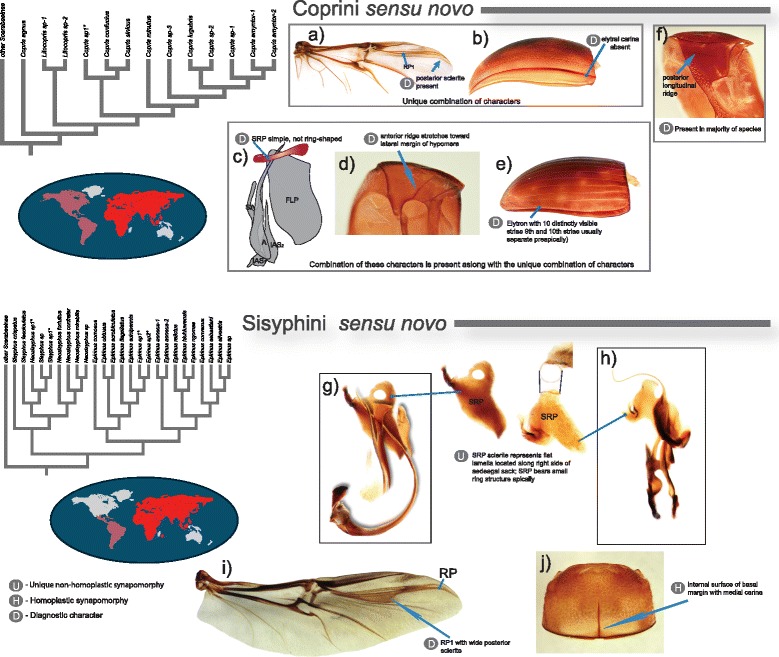
Illustrated synapomorphies and diagnostic characters defining Coprini *sensu novo* and Sisyphini *sensu novo*. Every synapomorphy or diagnostic statement is preceded by a grey circle indicating whether the synapomorphy is unique (U), homoplastic (H), or the statement is diagnostic (D). Explanatory text for character statements is shown next to the images in the figure; additional information is available in Table 1. Vein names are shown for some wing veins for annotation purposes. In some cases morphological parts of species from other tribes are used for illustration purposes. Phylogenetic trees refer to the representatives of the respective tribes from Fig. 3. Maps show the distribution of the tribes per biogeographic region; red color saturation corresponds to approximate species number. **a**). *Macroderes mutilans*; **b**). *Anachalcos convexus*; **c**). *Copris*; **d**). Scarabaeinae; **e**). *Copris sp.*; **f**). *Coptodactyla nitida*; **g**). *Epirinus sp.*; **h**, **i**, **j**).* Neosisyphus sp.*; **a**, **i**). wing; **b**, **e**). elytron; **c**, **g**, **h**). aedeagal sclerites; **f**, **j**). prothorax

The synapomorphies and diagnostic characters were identified based on the results from the recent global morphological phylogeny [1] and are provided in Figs. 4, 5 and Table 1. That morphological study covers all major dung beetle lineages and thereby is the best source for analyzing evolution of morphological characters in this group. Herein, the term synapomorphy refers exclusively to unambiguous synapomorphies which were identified in morphological phylogeny [1] by parsimony mapping of the morphological characters onto the selected most parsimonious tree (Fig. 6 in [1]). These synapomorphies can be classified into (i) non-homoplasious that uniquely identify clade and (ii) homoplasious that in addition to the clade of interest can identify some other clade.

**Table 1:** Synapomorphies and diagnostic characters defining new tribal concepts

Tribe Deltochilini (Canthonini) *sensu novo*:	
*U*	*101. Wing, RP*_*1*_ *posterior sclerite represents small basal appendix of RP*_*1.*_ Note: In *Anisocanthon* basal appendix of RP_1_ is reduced and poorly visible. In *Pseudocanthon* appendix of RP_1_ is separated from RP_1_.
*U*	*103. Wing, RA*_*4*_ *significantly thinner than RP*_*1*_ *, arcuate and not parallel to RA*_*4*_ *; RA*_*4*_ *fused basally with RP*_*1*_ *.*
Tribe Dichotomiini *sensu novo*:	
*H*	*13. Parameres, membrane on lower side strongly sclerotized with two notches basally.* Note: Investigation of additional material revealed that this character is absent in some Dichotomiini *sensu novo* which suggests a change of its status to at least a homoplastic synapomorphy and at the same time decreases the power of its diagnosability; therefore this character is not illustrated here.
*U*	*58. FLP sclerite elongated in frontal-rear plane usually small c-shaped.* Note: For readability purpose, the original character statement [1] was reworded.
*U*	*62. LC large, ring-shaped in horizontal plane.*
*H*	*66. Elytron with 8 distinctly visible striae.* Note: The number of visible striae is 8, the total number of striae is 9 as the last stria indistinctly bifurcates apically.
*H*	*105. Wing, posterior sclerite of RP*_*1*_ *separated from RP*_*1.*_
*U*	*123. Maxilla, stipital sclerite II with medial groove or its trace ; surface of groove usually shagreened.* Note: For readability purpose, the original character statement [1] was reworded.
*H*	*124. Galea, dorsal articular sclerite forms longitudinal carina on galea dorsal surface.* Note: In [1] this character represents a unique synapomorphy; however, it is absent in *Isocopris* (that was not included in [1]) that suggests a change of its status to at least a homoplastic synapomorphy.
*U*	*139. Epipharynx with triangular deep notch anteriorly.*
Tribe Sisyphini *sensu novo*:	
*U*	*50. SRP sclerite represents flat lamella located along right side of aedeagal sack; SRP bears small ring structure apically*
*U*	*86. Elytron, last stria (9* ^*th*^ *or 8* ^*th*^ *) visible at least preapically.* Note: For readability purpose, the original character statement [1] was reworded. We consider 8^th^ stria in *Neosisyphus* and 9^th^ in *Epirinus* to be homologous according to the criterion of position. This character reflects the degree of development of this stria. Since, the original statement, formulated for the needs of phylogenetic analysis, does not meet the needs of diagnosability, this character is not illustrated here but can be found in [1].
*H*	*162. Pronotum, internal surface of basal margin with medial carina.* Note: The degree of expression of this character varies within Sisyphini *sensu novo*.
*D*	*102. Wing, RP*_*1*_ *with wide posterior sclerite.* Note: Although this character does not represent an unambiguous synapomorphy in [1], it can be efficiently used for diagnostic purposes. In addition to Sisyphini *sensu novo* this character is also present in Onthophagini and Oniticellini.
Tribe Coprini *sensu novo*:	
*D*	*113. Wing, apical area bears sclerite located posteriorly of RP*_*1*_ *.*
*D*	*73. 1* ^*st*^ *elytral carina absent.*
*D*	*48. SRP simple not ring-shaped.*
*D*	*68. Elytron with 10 distinctly visible striae (9* ^*th*^ *and 10* ^*th*^ *striae usually separate preapically).* Note: For readability purpose, the original character statement [1] was reworded.
*D*	*157. Hypomera, anterior ridge stretches toward lateral margin of hypomera.*
*D*	*161. Hypomera, posterior longitudinal ridge present.*

This table lists synapomorphies and diagnostic characters defining the new tribal concepts. Number preceding character statement refers to the character number in [1]; capital letter indicates unique synapomorphy (U), homoplastic synapomorphy (H) and diagnostic character (D). The listed characters are illustrated in Figs. 4, 5 (except characters 13 and 86, see notes), additional information is provided in "Changes in classification" section

Diagnostic characters (e.g., in Coprini *sensu novo* and Sisyphini *sensu novo*) were elucidated using the character matrix of [1] by finding a unique combination of characters providing unequivocal diagnosis for the new concepts. Since Coprini *sensu novo* is not strictly monophyletic in [1] (see “Tribe Coprini *sensu novo* *”* chapter for discussion), its synapomorphies could not be assessed. Sisyphini *sensu novo* is characterized by both synapomorphies and one diagnostic character. We should note that diagnostic character might also be ambiguous synapomorphies.

Because the morphological phylogeny [1] includes only 37% of the global scarabaeine generic diversity, we manually investigated the presence of the potential diagnostic characters and putative synapomorphies in ~90% of all Scarabaeinae genera hitherto placed in Deltochilini, Dichotomiini and Coprini (see also Table 2).

**Table 2:** New tribal concepts and their genera

	Tribe/Genera	Inv.	Biogeographic Region
Tribe Sisyphini *sensu novo*			
1	*Epirinus* Reiche, 1841	M, P	Afrotropical
2	*Neosisyphus* Muller, 1942	M, P	Afrotropical, Oriental
3	*Nesosisyphus* Vinson, 1946	L	Mauritius
4	*Sisyphus* Latreille, 1807	P	Afrotropical, Oriental, Palaearctic, Neotropical
Tribe Dichotomiini *sensu novo*			
1	*Chalcocopris* Burmeister, 1846	M	Neotropical
2	*Dichotomius* Hope, 1838	M, P	Neotropical, Nearctic
3	*Holocephalus* Hope, 1838	S	Neotropical
4	*Isocopris* Pereira & Martinez, 1960	S	Neotropical
Tribe Coprini *sensu novo*			
1	*Copris* Muller, 1764	M, P	Afrotropical, Oriental, Palaearctic, Nearctic, Neotropical
2	*Litocopris* Waterhouse, 1891	P	Afrotropical
3	*Microcopris* Balthasar 1958	M	Oriental
4	*Pseudocopris* Ferreira, 1960	L	Afrotropical
5	*Pseudopedaria* Felsche, 1904	M	Afrotropical
Tribe Deltochilini (Canthonini) *sensu novo*			
1	*Anisocanthon* Martinez & Perreira, 1956	S	Neotropical
2	*Anomiopus* Westwood, 1842	M, P	Neotropical
3	*Canthon* Hoffmansegg, 1817	M, P	Neotropical, Nearctic
4	*Canthonidia* Paulian, 1939	S	Neotropical
5	*Canthotrypes* Paulian, 1939	S	Neotropical
6	*Deltepilissus* Pereira, 1949	S	Neotropical
7	*Deltochilum* Eschscholtz, 1822	M, P	Neotropical, Nearctic
8	*Eudinopus* Burmeister, 1840	P	Neotropical
9	*Hansreia* Halffter & Martinez, 1977	M, P	Neotropical
10	*Holocanthon* Martinez & Pereira, 1956	S	Neotropical
11	*Malagoniella* Martinez, 1961	M, P	Neotropical
12	*Megathopa* Eschscholtz, 1822	P	Neotropical
13	*Megathoposoma* Balthasar, 1939	M, P	Neotropical
14	*Melanocanthon* Halffter, 1958	S	Nearctic
15	*Pseudocanthon* Bates, 1887	S	Neotropical, Nearctic
16	*Scatonomus* Erichson, 1835	M	Neotropical
17	*Scybalocanthon* Martinez, 1948	M, P	Neotropical
18	*Scybalophagus* Martinez, 1953	M, P	Neotropical
19	*Sylvicanthon* Halffter & Marttinez, 1977	M	Neotropical
20	*Tetraechma* Blanchard, 1843	M	Neotropical
21	*Vulcanocanthon* Pereira & Martinez, 1960	S	Neotropical
22	*Xenocanthon* Martinez, 1952	S	Neotropical

List of genera assigned to the redefined tribes based on their new concepts. Column "Inv." (investigation source) specifies evidence based on which genus was attributed to the tribe. Abbreviations are as follows: (M) morphological phylogeny [1], (P) present phylogeny, (S) synapomorphies or diagnostic characters checked (material examined per genus is given in Additional file 24: Table S6), (L) synapomorphies or diagnostic characters were not investigated and genus was attributed based on description and overall similarity to the type genus of tribe

### Tribe Coprini sensu novo

Coprini Leach 1815: 96 (Coprides)

Type genus: *Copris* Geoffroy, 1762

#### Systematic note

Part of the genera of Coprini *sensu novo* (*Copris* and *Litocopris*) are monophyletic in the present molecular phylogeny. The global morphological phylogeny [1] reveals a polytomy of *Copris* with *Pseudopedaria + Micorcopris* (clade L4, Fig. 6 in [1]). The lack of resolution in Coprini *sensu novo* in morphology is the probable result of incomplete species sample from this tribe. It is noteworthy that genus *Microcopris* is considered by some authors [67] as a subgenus of *Copris* pointing out to their close relationship. In spite of lack of resolution, the present molecular results generally corroborate the morphological finding providing further evidence for the monophyly of Coprini *sensu novo*. However, more data are desirable to improve the support for this group.

In the present study Coprini *sensu novo* is well supported by ML and BI (Table 3); previous studies also suggest a separate monophyletic position for the members of Coprini *sensu novo* [1, 11, 22]. Coprini *sensu novo* comprises five genera (Table 2), all of which were members of the traditionally defined Coprini. We confidently place *Catharsius*, *Metacatharsius*, *Coptodactyla,* previously considered Coprini, outside Coprini *sensu novo* as neither molecules nor morphology support this placement. We transfer a few other Coprini genera *Thyregis, Synapsis, Copridaspidus* not included in present analyses to *incertae sedis* based on another morphological phylogeny [15] suggesting their sister relationships to the non-Coprini *sensu novo* genera *Coptodactyla, Heliocopris* and *Catharsius* correspondingly. All genera transferred to the *incertae sedis* category are listed in Additional file 23: Table S9.

**Table 3:** The support for the new tribal concepts in Scarabaeinae

Tribe\Dataset	ALL	G20	DT3	BI (G20)	Citations
Sisyphini *sensu novo*	81	77	58	1	[1, 66]
Dichotomiini *sensu novo*	83	78	51	1	[1, 10, 11, 19, 22]
Coprini *sensu novo*	100	100	100	1	[1, 11, 22]
Deltochilini *sensu novo*	43	49	44	1	[1, 10, 11, 19, 22]

Columns “ALL”, “G20” and “DT3” show bootstrap support for the new tribal concepts in ML analyses. Column “BI” shows Bayesian posterior probabilities for the analysis with G20 dataset. Column “Citations” lists publications which suggest similar tribal relationships

#### Diagnosis and synapomorphies

The lack of resolution in morphology makes identification of synapomorphies difficult for this tribe; therefore, here we provide only diagnostic characters aiding efficient identification of Coprini *sensu novo*. The tribe can be unequivocally differentiated by combination of two character states (Fig. 4 and Table 1): (i) apical area of wing bearing sclerite located posteriorly of RP_1_ and (ii) absent pre-epipleural (1st) elytral carina. In addition, species of Coprini *sensu novo* also share the following combination of character states: (i) SRP simple not ring-shaped, (ii) elytron with 10 distinctly visible striae and (iii) anterior ridge of hypomera stretches toward lateral margin of hypomera. The majority of genera, (except. *Microcopris,* for example), have hypomera with posterior longitudinal ridge; however, this character is present in other non-Coprini *sensu novo* genera as well (see character matrix in [1]).

#### Distribution

All five genera of Coprini *sensu novo* genera are primarily distributed in the Afrotropical and Oriental Regions. Some species of the type genus *Copris* are also found in North and Central America.

### Tribe Deltochilini (Canthonini) sensu novo

Deltochilini Lacordaire 1856: 78 (Deltochilides)

Canthonini van Lansberge 1874: 184 (Canthonides, type genus: *Canthon* Hoffmannsegg, 1817)

Scatonomini Lacordaire 1856: 87 (Scatonomides, type genus: *Scatonomus* Erichson, 1835)

Type genus: *Deltochilum* Eschscholtz, 1822

#### Systematic note

The traditional concept of the tribe Deltochilini (Canthonini) comprising 100+ genera was recovered highly polyphyletic by our results and previous studies [1, 10, 11, 22]. Deltochilini as traditionally defined form numerous monophyletic groups spread across the phylogenetic tree of Scarabaeinae. Both molecular results presented here (Table 3 for ML bootstrap and BI support) and morphological results [1] recover the monophyletic group of true Deltochilini (i.e. Deltochilini *sensu novo*) comprising the type genus of the tribe and allies, all of which exclusively occur in the New World. Morphological phylogeny recovered 11, while present molecular phylogeny recovered 10 genera in the clade Deltochilini *sensu novo*, 8 of those genera were shared between the two phylogenies (Table 2).

#### Diagnosis and synapomorphies

The morphological phylogeny suggests two unique and easily identifiable synapomorphies in wing venation (Fig. 4 and Table 1) characterizing the diagnosis of this new tribal concept. Investigation of morphology in genera traditionally assigned to Deltochilini and Dichotomiini allowed us to identify additional 9 genera that share the same synapomorphies with Deltochilini *sensu novo*. Based on this finding we assign these genera to the Deltochilini *sensu novo*. Beside those unique synapomorphies the general morphology of those 9 genera is similar to that of the genera included in our phylogenetic analyses. As a result, the new definition of Deltochilini limits its traditional concept by leaving only 22 genera out of 100+ within that tribe (Table 2).

#### Distribution

The majority of genera are distributed in the Neotropics, while some also occur in the Nearctic Region. Numerous other genera traditionally placed in Deltochilini from New World, all from Afrotropics, Oriental and Australasian Regions do not belong to Deltochilini *sensu novo* nor to any other known tribe given the results of aforementioned phylogenies; herein we treat them as *incertae sedis* (Additional file 23: Table S9).

### Tribe Dichotomiini sensu novo and the case of Ateuchini

Pinotini Kolbe 1905: 548 (Pinotinae)

Dichotomiini Pereira 1954:55

Dichotomides Halffter 1961:228

Dichotomiini Halffter and Matthews 1966: 256

Type genus: *Dichotomius* Hope, 1838 (*Pinotus* Erichson, 1847 is a junior synonym of *Dichotomius* Hope, 1838)

#### Systematic note

The name Dichotomiini has been hitherto considered unavailable as a family-group name due to the lack of description or validation [32] but nevertheless was widely used in Scarabaeinae. Thanks to our colleague (F. Vaz-de-Mello, CEMT) who gave a hint to previously overlooked publication [68], the name Dichotomiini has to be deemed available. According to the *International Code of Zoological Nomenclature* Article 13.2.1 [69] “a family-group name first published after 1930 and before 1961 … is available from its original publication only if it was used as valid before 2000 …”. The name Dichotomiini after its original publication in 1954 [68] was subsequently used before 2000 (e.g. [70]) which given the aforementioned Article confirms its availability.

Tribe Dichotomiini has been considered a junior synonym of Ateuchini [32] as the genus *Dichotomius* was deemed to be closely related to *Ateuchus* (the type genus for the tribe Ateuchini Laporte, 1840). Morphological phylogeny recovers that *Ateuchus* and allies (clade F1, Fig. 6 in [1]) are remotely related to *Dichotomius* lineage. Present molecular result recovers polyphyly of Ateuchini and suggests sister group relationship between *Dichotomius* and *Ateuchus + Scatimus*, while other representatives of Ateuchini subtribe Scatimina emerge as more remotely related. Although, molecular results support the monophyly of *Dichotomius + Ateuchus*, morphological analyses unequivocally point to their significant morphological divergence. In order to fulfill abovementioned classification principles, it is therefore convenient to separate *Dichotomius* + allies and *Ateuchus* + allies into two tribes. The main objective for following the splitting principle is to create the diagnosable groups. Thus, we split the tribe Ateuchini into two tribes Ateuchini and Dichotomiini *sensu novo*. In the present study Dichotomiini *sensu novo* is well supported by ML and BI (Table 3). The name Ateuchini has now to be applied only to the members of the genus *Ateuchus* and its close relatives (*sensu* [61]). The tentative list comprising 20 Ateuchini genera is given in Additional file 23: Table S9; however, the exact composition and diagnosis of this tribe requires additional investigation. The genera transferred from the traditional concept of Dichotomiini to *incertae sedis* category are also listed in Additional file 23: Table S9.

#### Diagnosis and synapomorphies

The monophyly of Dichotomiini *sensu novo* is well supported by molecules [11, 19, 22] as well as morphology [1, 10]. Based on recent morphological analyses [1] Dichotomiini *sensu novo* is defined by 4 unique and 4 homoplasious synapomorphies (Fig. 4, Table 1) which unequivocally diagnose this tribe.

#### Distribution

Dichotomiini *sensu novo* comprises four genera (Table 2) widespread in the Neotropics, of which *Dichotomius* is distributed in both Nearctic and Neotropical Regions.

### Tribe Sisyphini sensu novo

Sisyphini Mulsant 1842: 41 (Sisyphaires)

Type genus: *Sisyphus* Latreille, 1807

#### Systematic note

Genus *Epirinus* is found to be sister to the traditional Sisyphini genera *Sisyphus* and *Neosisyphus*. Present and previous [66] molecular as well as morphological [1] results strongly support this relationship, which suggests the transfer of *Epirinus* to Sisyphini. In present study Sisyphini *sensu novo* is well supported by ML and BI (Additional file 4: Table S3).

#### Diagnosis and synapomorphies

The diagnosis of expanded Sisyphini *sensu novo* is defined by two unique and one homoplasious synapomorphies (Fig. 4, Table 1).

#### Distribution

Sisyphini *sensu novo* comprises four genera, two of which *Sisyphus* and *Neosisyphus* primarily occur in the Afrotropical and Oriental Regions, some species of *Sisyphus* are also distributed in the Palearctic and the Neotropics. The distribution of the genus *Neosisyphus* is restricted to Mauritius Island, while *Epirinus* occurs in southern Africa.

## Conclusions

The present molecular phylogeny advances our knowledge on dung beetle relationships. We used these results in conjunction with the recent morphological phylogeny and evidences from molecular phylogenies that have been accumulated over the last decades to revise the concepts of three of the subfamily’s most problematic tribes (Deltochilini, Dichotomiini and Coprini). Although the result of the new classification proposed here leaves many dung beetle genera unclassified (*incertae sedis*), it creates a systematically based classification for the existing tribes and provides a clear direction for future work with these genera. At the same time deep relationships within the subfamily remain poorly supported pointing to the need of acquisition of additional data to resolve them. These issues have to be addressed by future studies aiming at integration of molecular, morphological and fossil data.

We propose that use of modern statistical methods for model adequacy evaluation has a potential to improve phylogenetic inference by detecting cases where substitution models do not perform well. Presently, data selection using this approach cannot be fully performed on big datasets due to computational constraints. The development of new software packages is needed to overcome this problem. At the same time it is noteworthy that inclusion of data where models do not adequately depict substitution process according to our analysis, did not substantially affect the final phylogenetic analyses. Likely the presence of strong signal in our dataset from large portions where the application of substitution models is plausible has compensated for the potential biases caused by the inclusion of partitions that were rejected in the adequacy assessments.

## Additional files

Additional file 1: Table S1. Accession numbers and vouchers information. (XLSX 64 kb)
Additional file 2: Table S2. List of genera with author citations. (XLSX 12 kb)
Additional file 3: Matrix S1. Data matrix used in the phylogenetic analyses. (NEX 3130 kb)
Additional file 4: Table S3. Primers used in the study. (DOCX 17 kb)
Additional file 5: Table S4. Partitions and their models used in the analyses. (DOCX 16 kb)
Additional file 6: Table S5. Data blocks and their *p*-values assessed using Bayesian posterior prediction in PuMA. (DOCX 17 kb)
Additional file 7: Statistics for MrBayes Runs. (ZIP 395 kb)
Additional file 8: Tree S12. Scarabaeinae ML tree of dataset ALL using at least 4 genes (including 18 s, 28sd2, 28sd3, CAD, 16 s, Tp1). (TREE 17 kb)
Additional file 9: Tree S13. Scarabaeinae ML tree of dataset DT3 using at least 5 genes (including 18 s, 28sd2, 28sd3, CAD, 16 s, Tp1, COI, CAD). (TREE 5 kb)
Additional file 10: Tree S1. Scarabaeinae ML tree of dataset ALL. (TREE 37 kb)
Additional file 11: Tree S2. Scarabaeinae ML tree of dataset G20. (TREE 37 kb)
Additional file 12: Tree S3. Scarabaeinae ML tree of dataset DT3. (TREE 37 kb)
Additional file 13: Table S7. Robinson-Fold distance and proportion of shared clades between ML analyses using datasets ALL, G20 and DT3 as well as BI with MrBayes using G20 dataset. (XLS 27 kb)
Additional file 14: Tree S4. MrBayes 50% majority consensus tree with support statistics. (TRE 270 kb)
Additional file 15: Tree S5. MrBayes 50% majority consensus tree with collapsed ambiguous branches (see Additional file 6 for details). (TRE 21 kb)
Additional file 16: Tree S6. POY Tree, estimated using parameter scheme 111 (see Methods section). (TRE 12 kb)
Additional file 17: Tree S7. POY Tree, estimated using parameter scheme 121 (see Methods section). (TRE 12 kb)
Additional file 18: Tree S8. POY Tree, estimated using parameter scheme 211 (see Methods section). (TRE 12 kb)
Additional file 19: Tree S9. POY Tree, estimated using parameter scheme 221 (see Methods section). (TRE 12 kb)
Additional file 20: Tree S10. POY Tree, estimated using parameter scheme 3211 (see Methods section). (TRE 12 kb)
Additional file 21: Tree S11. POY Tree, estimated using parameter scheme 3221 (see Methods section). (TRE 12 kb)
Additional file 22: Table S8. Bootstrap support in ML and posterior probabilities in BI analyses for the major inferred Scarabaeinae lineages. (XLSX 38 kb)
Additional file 23: Table S9. The list of Scarabaeinae genera attributed to the tribe Ateuchini and category *incertae sedis*. (XLS 40 kb)
Additional file 24: Table S6. Species used to check synapomorphies for the new tribal concepts. (DOCX 16 kb)
