# Supplementary material for: Multigene phylogenetic analysis redefines dung beetles relationships and classification (Coleoptera: Scarabaeidae: Scarabaeinae)
Source: BMC Evol Biol. 2016 Nov 29;16:257. doi: 10.1186/s12862-016-0822-x (PMC5129633; doi:10.1186/s12862-016-0822-x)
Supplement: Additional file 4: Table S3. — Primers used in the study. (DOCX 17 kb) [file 12862_2016_822_MOESM4_ESM.docx]

Supplementary material Table S3. Primers used in the study.

| **Genes** | **Primer name** | **Primer sequence (5'–3')** | **Reference** |
| --- | --- | --- | --- |
| 18s | 18s ai | CCT GAG AAA CGG CTA CCA CAT C | Whiting et al. (1997) |
|  | 18s bi | GAG TCT CGT TCG TTA TCG GA | Whiting et al. (1997) |
| 28s | D2F | AGT CGT GTT GCT TGA TAG TGC AG | Ocampo and Hawks (2006) |
|  | D2R | TTG GTC CGT GTT TCA AGA CGG G | Ocampo and Hawks (2006) |
|  | D3F | GAC CCG TCT TGA AAC ACG GA | Ocampo and Hawks (2006) |
|  | D3R | TCG GAA GGA ACC ACG TAC TA | Ocampo and Hawks (2006) |
| CAD | CD439F | TTCAGTGTACARTTYCAYCCHGARCAYAC | Wild and Maddison (2008) |
|  | CD688R | TGTATACCTAGAGGATCDACRTTYTCCATRTTRCA | Wild and Maddison (2008) |
|  | CD1098R2 | GCTATGTTGTTNGGNAGYTGDCCNCCCAT | Wild and Maddison (2008) |
| TP1 | TP675F | GAGGACCAAGCNGAYACNGTDGGTTGTTG | Wild and Maddison (2008) |
|  | TP643F | GACGATTGGAARTCNAARGARATG | Wild and Maddison (2008) |
|  | TP919R | GTCTCTTTGCGTYTTRTTRTADATYTTYTC | Wild and Maddison (2008) |
| Wg | Wg550F | ATGCGTCAGGARTGYAARTGYCAYGGYATGTC | Wild and Maddison (2008) |
|  | Wg578F | TGCACNGTGAARACYTGCTGGATG | Wild and Maddison (2008) |
|  | WgAbrZ | CACTTNACYTCRCARCACCARTG | Wild and Maddison (2008) |
|  | WgAbr | ACYTCGCAGCACCARTGGAA | Wild and Maddison (2008) |

**Supplementary References**

Ocampo F.C., Hawks D.C. 2006. Molecular phylogenetics and evolution of the food relocation behaviour of the dung beetle tribe Eucraniini (Coleoptera, Scarabaeidae, Scarabaeinae). Invertebrate Systematics. 20:557-570.

Whiting, M. F., Carpenter, J. C., Wheeler, Q. D., Wheeler, W. C. 1997. The Strepsiptera problem: phylogeny of the holometabolous insect orders inferred from 18S and 28S ribosomal DNA sequences and morphology. Systematic Biology 46: 1-68.

Wild A.L., Maddison D.R. 2008. Evaluating nuclear protein-coding genes for phylogenetic utility in beetles. Molecular Phylogenetics and Evolution. 48:877-891.
