# Supplementary material for: Multigene phylogenetic analysis redefines dung beetles relationships and classification (Coleoptera: Scarabaeidae: Scarabaeinae)
Source: BMC Evol Biol. 2016 Nov 29;16:257. doi: 10.1186/s12862-016-0822-x (PMC5129633; doi:10.1186/s12862-016-0822-x)
Supplement: Additional file 5: Table S4. — Partitions and their models used in the analyses. (DOCX 16 kb) [file 12862_2016_822_MOESM5_ESM.docx]

| **Partition #** | **Best Model** | **Included data blocks** |
| --- | --- | --- |
| **ML**  **Run #2** |  |  |
| 1 | GTR+G | loop regions of rDNA genes. |
| 2 | HKY+G | stem regions of rDNA genes. |
| 3 | GTR+G | COI-1 |
| 4 | GTR+G | COI-2 |
| 5 | GTR+G | COI-3 |
| 6 | SYM+G | CAD-A1; TP1-A1-B1; Wg-1-2. |
| 7 | SYM+G | CAD-A2-C1-D1. |
| 8 | GTR+G | CAD-A3-C3-D3. |
| 9 | GTR+G | CAD-B |
| 10 | GTR+G | CAD-C2-D2; Tp-A2-B2. |
| 11 | GTR+G | Tp-A3-B3; Wg-3. |
| **BI**  **Run #3** |  |  |
| 1 | GTR+G | loop regions of rDNA genes; COI-2. |
| 2 | HKY+G | stem regions of rDNA genes. |
| 3 | SYM+G | CAD-A1-A2-C1-D1; TP1-A1-B1; Wg-1-2. |
| 4 | GTR+G | CAD-A3-C3-D3. |
| 5 | GTR+G | CAD-B |
| 6 | GTR+G | CAD-C2-D2; Tp-A2-B2. |
| 7 | HKY+G | Tp-A3-B3; Wg-3. |

**Supplementary material Table S4. Partitions and their models used in the analyses**

The partitions with included data blocks used in maximum likelihood (ML) analyses (run #2) and Bayesian inference (BI) (run #3). Expansion of data blocks abbreviations is given in Supplementary material Table S5 and Fig. 1.
