# Supplementary material for: Multigene phylogenetic analysis redefines dung beetles relationships and classification (Coleoptera: Scarabaeidae: Scarabaeinae)
Source: BMC Evol Biol. 2016 Nov 29;16:257. doi: 10.1186/s12862-016-0822-x (PMC5129633; doi:10.1186/s12862-016-0822-x)
Supplement: Additional file 6: Table S5. — Data blocks and their p-values assessed using Bayesian posterior prediction in PuMA. (DOCX 17 kb) [file 12862_2016_822_MOESM6_ESM.docx]

Supplementary material Table S5. Data blocks and their p-values assessed using Bayesian posterior prediction in PuMA.

| **#** | **Gene** | **Partition with data blocks** | **Model** | **P-value from PuMA** |
| --- | --- | --- | --- | --- |
| 1 | 16s | Stems | GTR+I+G | 0.134807 |
| 2 | 16s | loops | HKY+I+G | 0.686163 |
| 3 | 18s | Stems | HKY+I+G | 0.429387 |
| 4 | 18s | loops | HKY+I+G | 0.371612 |
| 5 | 28sd2 | Stems | GTR+I+G | 0.094151 |
| 6 | 28sd2 | loops | K80+I+G | 0.0262172 |
| 7 | 28sd3 | Stems | HKY+I+G | 0.151926 |
| 8 | 28sd3 | loops | F81+I+G | 0.092725 |
| 9 | CAD | A1;A2 | K80+I+G | 0.0692883 |
| 10 | CAD | A3;C3;D3 | HKY+I+G | 0.768902 |
| 11 | CAD | B123 | HKY+G | 0.236091 |
| 12 | CAD | C1;D1 | GTR+G | 0.019971 |
| 13 | CAD | C2 | K80+I+G | 0.2222222 |
| 14 | CAD | D2 | JC | 0 |
| 15 | COI | Codon1 | GTR+I+G | 0.000713 |
| 16 | COI | Codon 2 | GTR+I+G | 0.723252 |
| 17 | COI | Codon3 | HKY+G | 0.982168 |
| 18 | Tp1 | A1;B1 | GTR+I+G | 0 |
| 19 | Tp1 | A2;B2 | GTR+I+G | 0.029957 |
| 20 | Tp1 | A3;B3 | SYM+I+G | 0.016405 |
| 21 | Wg | Codon1 | SYM+G | 0.127675 |
| 22 | Wg | Codon2 | K80+I+G | 0.089263 |
| 23 | Wg | Codon3 | HKY+G | 0.614836 |

Column “Partition with data blocks” lists partitions and their *a priori* data blocks (separated by semicolon). The assignment of data blocks to the partitions was done using Partition Finder. The genes were split into *a priori* data blocks based on secondary structure (rDNA genes), codon position (COI and Wg) or codon position and domain structure (CAD, Tp1). The domain structure for CAD and Tp1 as well as the graph of p-values are illustrated in Figure 1. The abbreviations for domains are as follows CAD: (A) Class I glutamine amidotransferase-like, (B) DNA identified as not falling in any domain (this region is not split by codons), (C) PreATP-grasp domain, (D) PreATP-grasp domain; Tp1: (A) DNA breaking-rejoining enzymes, (B) DNA breaking-rejoining enzymes.
