# Supplementary material for: Multigene phylogenetic analysis redefines dung beetles relationships and classification (Coleoptera: Scarabaeidae: Scarabaeinae)
Source: BMC Evol Biol. 2016 Nov 29;16:257. doi: 10.1186/s12862-016-0822-x (PMC5129633; doi:10.1186/s12862-016-0822-x)
Supplement: Additional file 7: — Statistics for MrBayes Runs. (ZIP 395 kb) [file 12862_2016_822_MOESM7_ESM.zip › Settings and Statistics for MrBayes Runs/Appendix_1-Settings and Statistics for MrBayes Runs.docx]

Online Appendix 1. Settings and Statistics for MrBayes Runs

Settings

We ran MrBayes with default priors except for the branch length. The default exponential branch length prior is known to cause bias in the branch length estimates in partitioned datasets [1, 2]. We used the compound Dirichlet prior instead as suggested by [3] and [2] with the following parameters *GammaDir (αT=1, βT=0.034, α=1, c=1)*. The *αT*=1 was chosen following [3], while *βT*=0.034 was calculated using tree length estimates from the ML analyses in order to conjugate prior distribution with likelihood. Due to the large size of the present dataset the following strategy was employed to run the Bayesian analyses. First, for each dataset (G20 and DT3) we ran 10 separate runs with a single cold chain and switched Markov Chain Monte Carlo (MCMC) diagnostics (*nchains=1, mcmcdiagn=no*) for 20M generations. The seeds for these runs were drawn at random from a uniform distribution (from 0 to (1*10^10)-1) in order to initiate runs from widely dispersed starting points. Sampling from the posterior distribution using only cold chain with MCMC diagnostics switched off is computationally significantly faster, although is not as effective as coupled chain in exploration of posterior space. Given the large size of the dataset we preferred running a large number of generations rather than using computationally slow coupled chains. After 20M generations we checked the convergence of the 10 runs visually in Tracer [4]. The MCMC chain trace in all runs of the DT3 dataset demonstrated very poor mixing; therefore we excluded it from the further analyses. All runs of dataset G20 converged to somewhat similar points in the posterior space. We selected two runs from this dataset which produced the highest likelihood scores. These runs were allowed to run for an additional 60M generations, sampling parameters every 1000^th^ generation. Afterwards we compared runs and found that the mixing of the parameter sample in one run was better than that in the other and so we tuned the latter run, except for the tree, with the parameters taken from the superior run. In total we ran these two runs for 180M generation (ca. 4 weeks), standard deviation of split frequencies (sdsf) achieved 0.077 that was slightly higher than the minimum acceptable value of 0.05. We further investigated the statistics for the posterior samples of these two runs and found that they provide a reasonable topological sample that is sufficient for constructing a stable consensus tree (see below).

Results

The two final MrBayes runs ran for 180M generations and generally converged after 150M generations, which were discarded as burn in. The average estimated sampled size for all parameters was greater than the minimal recommended value of 100 and the potential scale reduction Factor (PSRF) approached 1.0 as suggested in [5]. Average standard deviation of split frequencies was 0.077 which is slightly higher than the acceptable value of 0.05 and the distribution of likelihood values for the two runs did not precisely overlapped (online Appendix 1, Fig. 1d) pointing to differences in the topological sampling. We investigated additional statistics for the posterior sample to elucidate if 50% Bayesian majority consensus tree from the two runs can be considered stable in terms of topology and posterior support. 90.5% of splits which were sampled by both runs have standard deviation of posterior probability less than 0.01 between the runs (online Appendix 1, Fig. 1e). Of the remaining splits, which were recovered in either one of two runs but not both, only 18% contributed the most to the topological differences between the runs. These more disruptive splits had significantly lower posterior probabilities than those recovered in both runs (online Appendix 1, Fig. 1f).

The MCMC sampling of topology is the hardest issue in MrBayes [6] and is generally ineffective at sampling posterior distributions in big datasets [7, 8]. Given the high topological variance for the current dataset (95% credible set contains 1901 trees) and its big size, the splits with low posterior probability seem significantly more difficult to sample in comparison to the splits with high probability. Therefore, based on these empirical results, we suggest that two runs adequately sampled splits with high probability but did not perform well with regards to those with low probability. Splits with higher probability contribute the most to the topology and support of the Bayesian majority consensus tree. The splits of this consensus have low sdsf between the runs with 96.5% of them sharing sd < 0.01 (online Appendix 1, Fig. 1e, red line) and average sdsf 0.013, therefore this consensus tree (online Appendix 1, Fig. 1a-c; Tree S10) inferred by the two runs can be considered stable. Since in this study we are primarily interested in topological pattern of the dung beetle phylogeny, we assume that the present Bayesian consensus tree archives our goal.

The possibility to achieve better convergence between the runs through longer run time or coupled chains (e.g. 4 coupled chains decreases computation by a factor of 6), it is not guaranteed. However, this undoubtedly requires a significantly longer runtime (e.g., at least few months), which is impractical. Since we found evidence that stable consensus was achieved we did not continue with further MrBayes analyses.

To improve the credibility of the Bayesian consensus tree we collapse those branches where standard deviation between runs can significantly affect the occurrence of split in consensus tree (i.e. [posterior probability] minus [sd between runs that is < 0.5]). The consensus tree had a few such branches (herein called ambiguous) which are colored red in Figure 1a. The tree with these branches collapsed is shown in Figure 1b, c and is also provided as a tree file (Tree S11). The sdsf in this tree is low (online Appendix 1, Fig. 1d), while the posterior support is high (online Appendix 1, Fig. 1c, g).

A wide range of different model options in MrBayes makes this program perfect for BI in general; however, MrBayes is slow computationally when it comes to big, especially nucleotide, datasets. Distribution of different MCMC chains between processors – the only parallelization procedure efficient for nucleotide data in MrBayes – does not take full advantage of supercomputer resources [9]. Alternatively, ExaBayes the program that uses advanced computational techniques for BI of big datasets on computer clusters implements only exponential branch length priors which was shown to bias branch length estimation in partitioned datasets [1, 2]. The use of the unpartitioned dataset does not seem plausible biologically in our study, which currently limits implementation of ExaBayes.

References

1. Brown JM, Hedtke SM, Lemmon AR, Lemmon EM: **When trees grow too long: investigating the causes of highly inaccurate Bayesian branch-length estimates**. *Systematic Biology* 2010, **59**(2):145-161.

2. Zhang C, Rannala B, Yang Z: **Robustness of compound Dirichlet priors for Bayesian inference of branch lengths**. *Systematic biology* 2012.

3. Rannala B, Zhu T, Yang Z: **Tail paradox, partial identifiability, and influential priors in Bayesian branch length inference**. *Molecular biology and evolution* 2012, **29**(1):325-335.

4. **Tracer v1. 6.** [**http://beast.bio.ed.ac.uk/Tracer**](http://beast.bio.ed.ac.uk/Tracer) [ <http://beast.bio.ed.ac.uk/Tracer>]

5. Gelman A, Rubin DB: **Inference from iterative simulation using multiple sequences**. *Statistical science* 1992, **7**(4):457-472.

6. Lakner C, Van Der Mark P, Huelsenbeck JP, Larget B, Ronquist F: **Efficiency of Markov chain Monte Carlo tree proposals in Bayesian phylogenetics**. *Systematic biology* 2008, **57**(1):86-103.

7. Bouchard-Côté A, Sankararaman S, Jordan MI: **Phylogenetic inference via sequential Monte Carlo**. *Systematic biology* 2012, **61**(4):579-593.

8. Hackett SJ, Kimball RT, Reddy S, Bowie RC, Braun EL, Braun MJ, Chojnowski JL, Cox WA, Han K-L, Harshman J: **A phylogenomic study of birds reveals their evolutionary history**. *science* 2008, **320**(5884):1763-1768.

9. Aberer AJ, Kobert K, Stamatakis A: **ExaBayes: Massively Parallel Bayesian Tree Inference for the Whole-Genome Era**. *Molecular biology and evolution* 2014, **31**(10):2553-2556.

10. Revell LJ: **phytools: an R package for phylogenetic comparative biology (and other things)**. *Methods in Ecology and Evolution* 2012, **3**(2):217-223.

Figure Caption

Online Appendix 1, Figure 1. Bayesian majority consensus (50%) trees and statistics for two runs in MrBayes.

**a).** Bayesian majority consensus (50%) tree from two runs (Tree S10), with branches [posterior probability] minus [sd between runs that is < 0.5] colored in red (branches colored using R package Phytools v. 0.4-45 [10]); **b).** Bayesian majority consensus (50%) tree from two runs (Tree S11), with branches [posterior probability] minus [sd between runs that is < 0.5] collapsed, color of branches corresponds to the value of average standard deviation of split frequencies between two runs (branches colored using R package Phytools v. 0.4-45 [10]); **c)**. Bayesian majority consensus (50%) tree from two runs (Tree S11), with branches [posterior probability] minus [sd between runs that is < 0.5] collapsed, color of branches corresponds to the posterior probability value; **d).** Distribution of likelihood values for two runs in MrBayes; **e).** Empirical cumulative density function of standard deviation of split frequencies (sdsf) for the splits with average probability > 0.5 (ecdf red line, splits are shown in the consensus tree (online Appendix 1, Fig. 1a)) and for the splits found in all two runs (blue line); **f).** Distribution of the posterior probability for splits sampled only by one out of two runs (red) and by all two runs (blue); **g).** Distribution of the posterior probability for splits (tree in online Appendix 1, Fig. 1b, c) when ambiguous branches (colored red in online Appendix 1, Fig. 1a) are collapsed.
