# Supplementary figures and images for: Multigene phylogenetic analysis redefines dung beetles relationships and classification (Coleoptera: Scarabaeidae: Scarabaeinae)
Source: BMC Evol Biol. 2016 Nov 29;16:257. doi: 10.1186/s12862-016-0822-x (PMC5129633; doi:10.1186/s12862-016-0822-x)

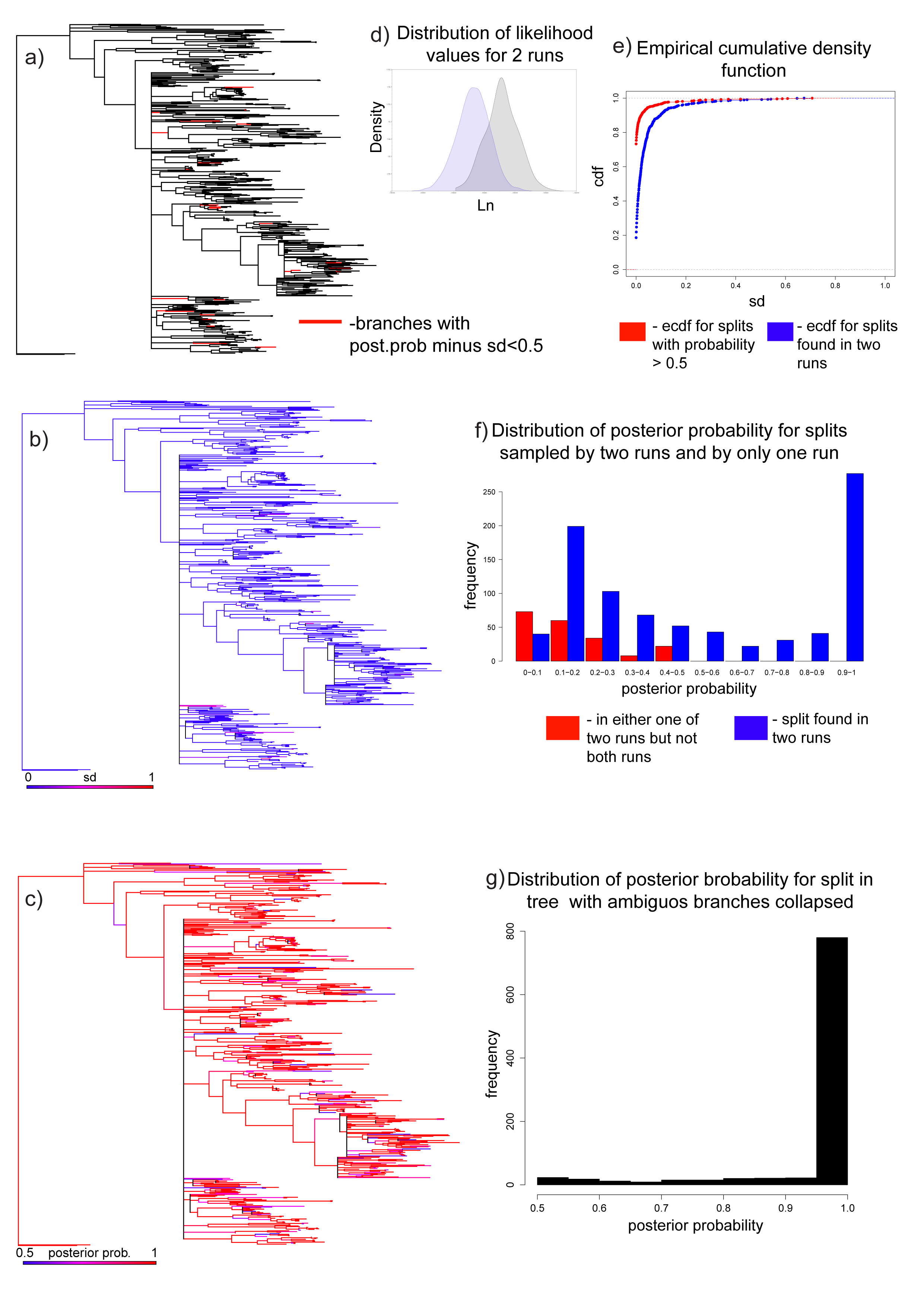

Supplement: Additional file 7: — Statistics for MrBayes Runs. (ZIP 395 kb) [file 12862_2016_822_MOESM7_ESM.zip › Settings and Statistics for MrBayes Runs/Fig_1-Appendix_1.tif]
