# Supplementary material for: Multigene phylogenetic analysis redefines dung beetles relationships and classification (Coleoptera: Scarabaeidae: Scarabaeinae)
Source: BMC Evol Biol. 2016 Nov 29;16:257. doi: 10.1186/s12862-016-0822-x (PMC5129633; doi:10.1186/s12862-016-0822-x)
Supplement: Additional file 24: Table S6. — Species used to check synapomorphies for the new tribal concepts. (DOCX 16 kb) [file 12862_2016_822_MOESM24_ESM.docx]

Supplementary material Table S7. Species used to check synapomorphies for the new tribal concepts.

| **Species** | **New tribal concept** |
| --- | --- |
| *Holocephalus sp.* | Pinotini |
| *Isocopris inhiatus* (Germar, 1824) | Pinotini |
| *Anisocanthon villosus* (Harold 1868) | Deltochilini |
| *Canthonidia rubromaculata* Blanchard 1846 | Deltochilini |
| *Melanocanthon punctaticollis* Schaeffer 1915 | Deltochilini |
| *Pseudocanthon perplexus* (Leconte 1847) | Deltochilini |
| Deltepilissus infernalis (Harold, 1880) | Deltochilini |
| *Xenocanthon vianai* Martinez, 1952 | Deltochilini |
| Vulcanocanthon seminulum (Harold, 186) | Deltochilini |
| Holocanthon mateui Martinez & Pereira, 1956 | Deltochilini |
| Canthotrypes oberthueri Paulian, 1939 | Deltochilini |

See Supplementary material Table S6 for details.
